# Randomized controlled pilot study of an app-based intervention for improving social skills, face perception, and eye gaze among youth with autism spectrum disorder

**DOI:** 10.3389/fpsyt.2023.1126290

**Published:** 2023-04-27

**Authors:** Kyongmee Chung, Eunsun Chung

**Affiliations:** Digital Mental Health Laboratory, Department of Psychology, Yonsei University, Seoul, Republic of Korea

**Keywords:** autism spectrum disorder, technology-based intervention, eye gaze, face perception, social skills

## Abstract

**Introduction:**

This pilot study aimed to examine the effectiveness of an app-based intervention (Yface) in 53 children with autism spectrum disorder. Yface is a combined program that improves social skills, facial perception, and eye gaze.

**Methods:**

Children were randomly assigned to one of the two training groups or a waitlist control group. One of the training groups completed the Yface training program lasting for 66 days, while the other training group used a similar app called Ycog, which focuses on cognitive rehabilitation. Questionnaires, computerized tasks, and semi-structured interviews were administered to children and their parents at pre- and post-training sessions.

**Results:**

The Yface group showed improvements in face perception and some social skills compared to waitlist controls, and in eye gaze compared to the Ycog group.

**Discussion:**

Our results suggest that this app-based intervention is effective in improving targeted social skills and face perception, although their relative effectiveness differs across skill domains.

## 1. Introduction

Autism spectrum disorder (ASD) is a neurodevelopmental disorder characterized by persistent deficits in social interaction and communication across multiple contexts, as well as repetitive and restricted patterns of behaviors, interests, or activities (1). Impairments in social interaction are typically manifested in limited use or detection of non-verbal social cues (2) and a lack of social–emotional reciprocity (3). Depending on the theoretical orientation, each intervention takes a different approach to enhancing the social ability of those with ASD.

The most common method for improving social ability among children with ASD is social skills training (SST) (4, 5). SST follows the principles of applied behavior analysis (ABA^1^) and focuses on teaching specific social skills appropriate for each developmental age, with the assumption that social impairments are caused by a lack of acquisition of these skills. Sample skills include social initiation (8), joint attention (9), and communication skills (10). SST is typically provided *via* face-to-face instruction in either individual or group formats (4). Despite its established effectiveness, its use in real-life settings is rather limited because of high costs, a lack of professionals, and restricted accessibility (11, 12).

Two other approaches to improving social ability have been noted, and one of them targets the perceptual process on the assumption that deficits in the perception of faces are the key feature of social deficits (13, 14). Weak central coherence is a well-established hypothesis explaining the mechanism behind limited face perception among individuals with ASD (15), who show superior local and/or inferior global processing, resulting in difficulties in integrating contextual information into a meaningful whole (16, 17). Examples include superior performance of ASD groups over typically developing children in an embedded figures task (18), block design tests (19), and face inversion tasks (20).

However, some hypothesize that social deficits among individuals with ASD may be the cause of their reluctance to make eye contact (21), given that it is a critical part of everyday social interactions and, lacking this ability is related to difficulties in social relationships (22). Several independent research groups (23, 24) have empirically supported the eye contact hypothesis among individuals with ASD, as shown in the study by Klin et al. (25), which reported unusual eye contact and excessive focus on the mouth area. Individuals with ASD show reduced fixation on the eye region of the face (26), are slower at detecting facial changes (27), and spend less time fixating on other faces (28). Despite the argument that this indifference may be caused by contextual factors rather than perceptual deficits (29), the atypical eye contact hypothesis still receives attention as an explanation for social deficits in ASD.

Unlike SST, however, efforts to develop training programs based on hypotheses assuming deficits in face perception and eye contact have not been actively pursued until recently, mainly because training to change perceptual and eye contact patterns requires extensive and repeated practice; hence, it is not feasible in terms of cost and time in face-to-face interventions. Technology-based intervention, a new treatment delivery method that provides psychological treatment *via* mobile application, has received significant attention due to its high accessibility and cost-effectiveness, compared to traditional face-to-face treatment (30, 31). It became more popular after 2017, when the US Food and Drug Administration approved its first prescription of digital therapeutics, the reSET (peartherapeutics.com), and following the tremendous increase in demand for non-face-to-face psychological intervention after the COVID-19 pandemic. Mobile applications have been extensively pursued both in the academic and business fields as an assisted, if not alternative, intervention method for diverse mental disorders.

Owing to recent technical advances in computers and mobile devices, where face stimuli can be presented in diverse ways and training records are easily traceable, several training programs have been developed. For example, Faja et al. (32) administered computer-based training for holistic face processing to 10 individuals with ASD, and reported improved sensitivity in face discrimination only in the training group. Tanaka et al. (33) developed a face processing/facial recognition training program and demonstrated its effectiveness in 79 children with ASD over 20 h. Recently, Oh and Chung (34) reported a significant improvement in facial recognition and eye gaze among 33 children with ASD after using a computer-based eye contact and holistic face processing training program for 10–15 min per day over 66 days.

A few attempts have been made to develop a technology-based program that combines tasks for improving face perception, eye gaze, and SST programs. For example, the Junior Detective Training Program developed by Beaumont and Sofronoff (35) is a computer game that combines training skills for coping with social situations and perceiving complex facial emotions. Improved social skills, emotion recognition, and problem solving were observed in the training group compared to the waitlist control group, and these results were maintained at the 5-month follow-up. A computer-based intervention called “FaceSay” consists of a set of games that train specific social skills in addition to eye gaze and recognition of facial identity and emotion (36). FaceSay was administered to 49 children with ASD over 6 weeks. The authors reported improved facial identity recognition, emotion recognition, and positive social interaction with peers in the training group compared to the waitlist control group. This result was replicated in a study by Rice et al. (14) in which 31 children with ASD were assigned to either a training or waitlist control group, with the training lasting for 10 weeks. Chung et al. (37) conducted a pilot study for a mobile application called “Look at Me”^2^ that combined face recognition and SST. This program was applied to 28 children with ASD using a one-group pre- and post-test design, and improvement in social responsiveness was observed after 8 weeks of training.

These studies have shown the applicability and potential of a combined training program for the above-mentioned hypotheses (social skills, face perception, and eye gaze), but there are some limitations. First, most of these programs include only a few tasks (e.g., three tasks in FaceSay) without balancing the task type across training areas (36). Second, three out of four studies adopted a waitlist control group as a comparison group, and one study did not even have a control group. The waitlist control group is criticized as weak, requiring careful interpretation of any positive results because of the belief that any treatment is better than no treatment (14). Third, these studies generally used subjective measures, mainly parent proxy self-reports, as the dependent variables. The use of multimodal assessment instruments, including both subjective and objective measures, is recommended in any treatment outcome study (38). This criticism should be taken seriously and addressed appropriately when testing the effectiveness of a combined program.

This randomized controlled pilot study aimed to test the effectiveness of an app-based training program in increasing social ability by combining training in social skills, face perception, and eye gaze among children and adolescents with ASD.

## 2. Methods

### 2.1. Participants

**Figure 1:**
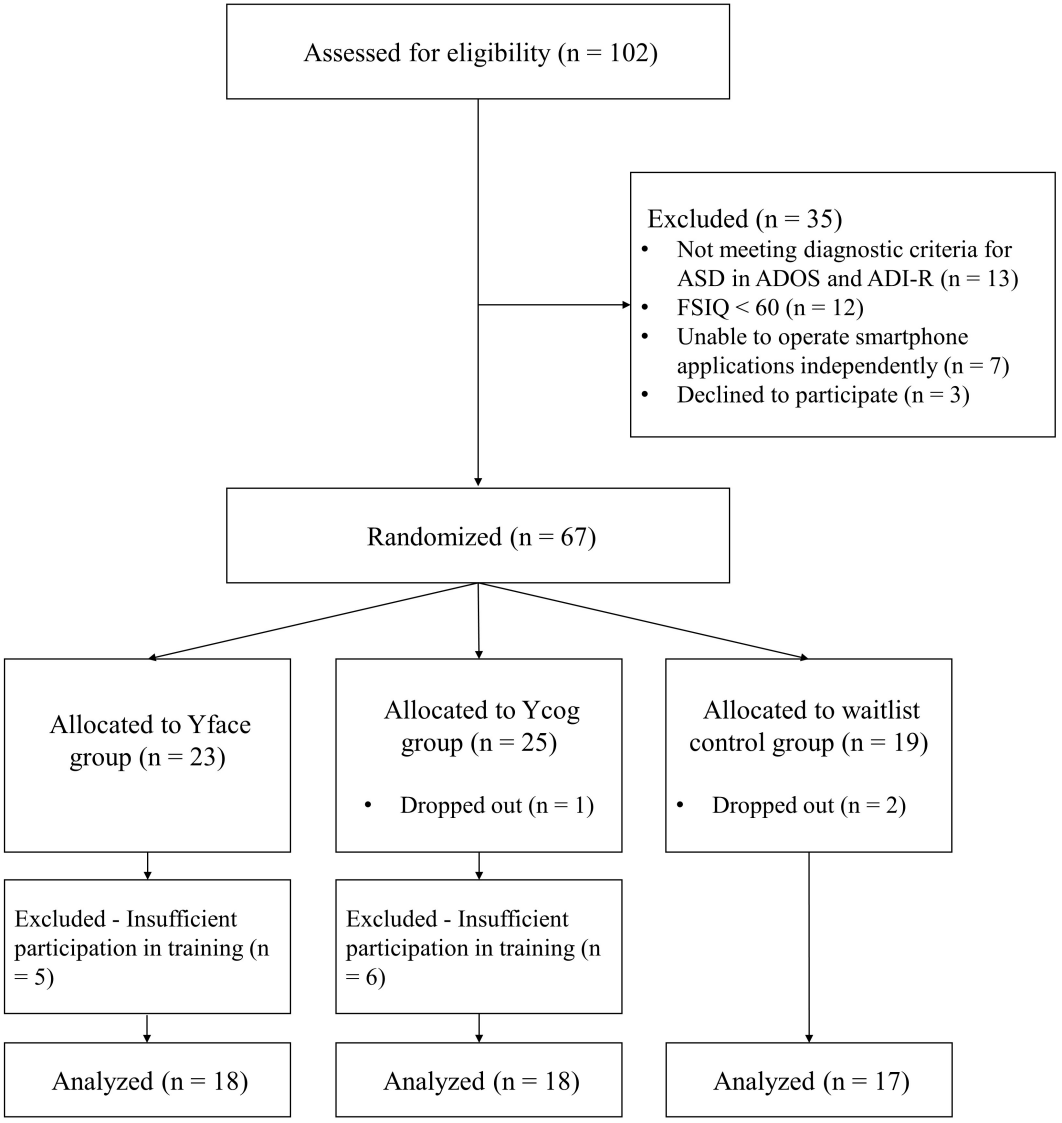
Flowchart of the study. ASD, autism spectrum disorder; ADOS, autism diagnostic observation schedule; ADI-R, autism diagnostic interview-revised; FSIQ, full-scale IQ.

Participants were recruited *via* advertisements on several internet sites for parents of children with ASD, posts in social welfare agencies in the Seoul Metropolitan area, and email announcements to special education teachers and mental health professionals specializing in developmental disorders. A total of 102 children aged 7–15 years and their parents were contacted, and those children who fulfilled the following inclusion criteria in the screening process were included in this study: (1) met the diagnostic criteria for ASD in the Autism Diagnostic Observation Schedule (ADOS) and the Autism Diagnostic Interview-Revised (ADI-R), administered by the research team; (2) scored ≥60 on the Full-Scale IQ (FSIQ) administered using the fourth edition of the Korean–Wechsler Scale of Intelligence (K-WISC-IV); (3) were able to independently operate smartphone applications; and (4) consented to participate (child and parent; Figure 1).

Thus, 67 children were matched for age and intelligence, and were randomly assigned to the Yface group, Ycog group, a comparison training group that focuses on executive function training, or waitlist control groups. Of these, three children dropped out of the study; two refused to visit the research site in the post-test due to difficulties in traveling a long distance, and one failed to complete the training despite prompts. In the analysis stage, an additional 11 children who completed <80% of the training were excluded from the data analysis. As a result, the study was completed with a final sample of 53 participants (*M* = 10.74 years, SD = 2.88, boys = 51, girls = 2). Information on the sex, age, and IQ of each group is presented in Table 1. There were no significant group differences in age or IQ. All procedures were approved by Yonsei University Institutional Review Board (no. 7001988-201712-SB-253-10).

**Table 1:** Distribution of sex, age, and IQ of participants by group.

Category	Participants (*N* = 53)					
	Yface group (*n* = 18)	Ycog group (*n* = 18)	Waitlist control (*n* = 17)	*F*	*df*	*p*-value
Sex (male/female)	18/0	17/1	17/0			
Mean age (*SD*)	11.61 (2.97)	10.28 (2.82)	10.29 (2.78)	1.275	2	0.288
Mean IQ (*SD*)	88.94 (19.54)	82.44 (15.36)	78.17 (15.67)	1.790	2	0.178

IQ, intelligence quotient; SD, standard deviation.

### 2.2. Measures

#### 2.2.1. Korean–Wechsler scale of intelligence

The K-WISC-IV (39) was used to assess participants’ intellectual ability. The research team administered the K-WISC-IV under the training and supervision of a licensed psychologist to generate the FSIQ. The examination took ~60–90 min.

#### 2.2.2. Autism diagnostic observation schedule and the autism diagnostic interview-revised

The Korean versions of the ADOS (40) and ADI-R (41) were used to verify the participants’ autism diagnosis. The ADOS is a semi-structured assessment instrument for diagnosing ASD (42). The ADI-R is a standardized semi-structured clinical diagnostic interview for caregivers of people with ASD (43). In this study, these instruments were administered by trained researchers under the supervision of the first author, a clinical psychologist with ADOS and ADI-R research certification. Depending on the participant, the ADOS took ~30 min to an hour, and the ADI-R took ~2–3 h.

#### 2.2.3. Social responsiveness scale

The SRS was used to measure the severity of social symptoms (44). The Korean version of the SRS was obtained from the Western Psychological Services *via* email, and the SRS scale was purchased from the WPS website. This scale comprises five sub-areas, and uses a 4-point scale from “*not true*” (0 points) to “*always almost true*” (3 points). The total score ranges from 0 to 195 points, with higher scores indicating lower levels of social responsiveness and social interaction. Raw scores, instead of T-scores, were used in this study because this scale has not been standardized for the Korean population. The internal consistency (Cronbach’s *α*) was 0.93 in Constantino et al. (44) and in this study as well.

#### 2.2.4. Semi-structured interview on social skills, face perception, and eye gaze

A modified version of the semi-structured interview developed by Oh and Chung (34) was administered to examine the participants’ social skills, face perception, and eye gaze in daily life. The interview questionnaire covered three areas: social skills and face perception (e.g., “Does the child recognize the face of a person consistently in various situations [e.g., the person in a cap, the same person in a picture]?”) and eye gaze (e.g., “How often does the child make eye contact with the caregiver?”). Social skills items were generated from previous literature that measured the ability to identify social cues and act appropriately. They included verbal communication (e.g., “Does the child take turns with the caregiver on general topics [e.g., the weather, vacations]?”) (45), non-verbal communication (e.g., “Does the child detect social cues in conversation [e.g., yawning]?) (2), and interpersonal relationships (e.g., “Does the child express its interest through behavior when peers are playing around it?”) (46). Content validity was then checked by three Board Certified Behavior Analysts (BCBAs). The final items were proofread by two elementary school Korean language teachers and a Korean linguistics expert with a doctoral degree. Due to the absence of an assessment instrument that included measures for eye gaze, face perception, and social skills, a semi-structured interview was developed by the research team to meet this need.

The final version of the semi-structured interview comprised 58 items across three areas, and was administered by a trained interviewer who asked the questions and recorded the responses directly from each parent. The interviewer assessed the frequency and appropriateness of each child’s behavior on a 7-point Likert-type scale. The interview took ~30 min to complete. The internal consistency scores (Cronbach’s *α*) for social skills, face perception, and eye gaze were 0.87, 0.93, and 0.92, respectively.

### 2.3. Computerized task

#### 2.3.1. Dot-probe task

**Figure 2:**
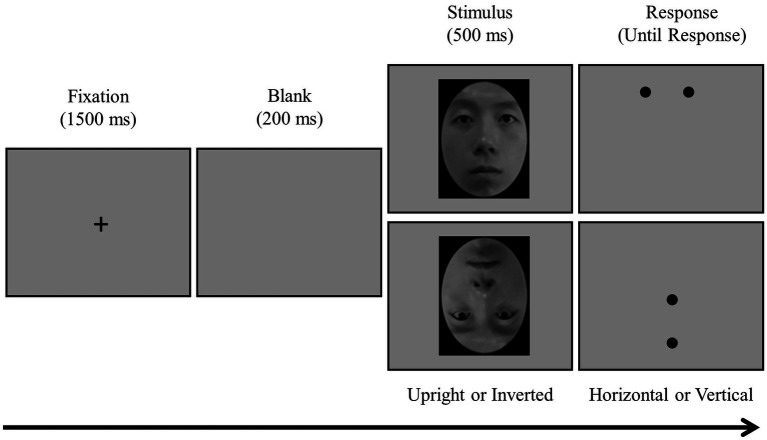
Dot-probe task procedure.

To measure attentional bias for eyes, a modified version of the dot-probe task (47) was developed and administered. In the task, after a fixation cross (+) was presented in the middle of the screen, either an upright or an inverted face stimulus was presented at random (Figure 2). After the stimulus disappeared, two dots appeared (aligned either horizontally or vertically) at the place where the eyes or the mouth were located before, and participants were instructed to respond quickly about whether the dots were horizontally or vertically aligned using a keyboard with alphabet stickers (horizontal = “S” and vertical = “L”). A total of 160 main trials were administered randomly: 40 trials in each of the four conditions in a 2 (upright or inverted face) × 2 (eye or mouth) design. The task took ~15 min to complete.

The dependent variable was the attentional bias score for eyes: attentional bias score = average reaction time when a dot was presented on the mouth – average reaction time when presented on the eyes (48). The assumption was that the reaction time would be faster for dots in the location of the eyes if participants showed attentional bias for the eyes, while the reaction time would be slower if participants showed attentional bias for some other areas (especially the mouth). Therefore, greater differences in reaction time indicated a higher level of attentional bias for the eyes.

### 2.4. Design and procedure

#### 2.4.1. Experimental design

A 2 × 3 group design was applied: two time points (pre- and post-intervention) as within-participant variables and three intervention groups (Yface, Ycog, and waitlist control) as between-participant variables.

#### 2.4.2. Procedure

This study was conducted in the following order: screening, pre-intervention assessment, intervention, and post-intervention assessment. Parents who agreed to participate in the study visited the laboratory thrice with their children for screening, pre-intervention assessment, and post-intervention assessment. On the first visit, written informed consent was obtained, and the K-WISC-IV, ADOS, and ADI-R were administered by graduate students in clinical psychology under the supervision of the corresponding author.^3^ Participants who met the inclusion criteria were randomly assigned to one of the three groups. The K-WISC-IV, ADOS, and computerized tasks were administered to the children, while the parents completed the ADI-R, SRS, and semi-structured interviews.

For both the Yface and Ycog groups, after completing the pre-intervention assessments, the parents downloaded the application on their smartphones with the help of the research team, and received a manual booklet. In light of previous research showing that at least 66 days of training was needed for behavior change (49), each participant was instructed to complete six games every day for the next 66 days. The progress of all participants was monitored by a designated research team member using a dashboard software program. Participants who did not complete the games at least three times per week were contacted through a call or text message. Approximately 38% (Yface) and 35% (Ycog) of the participants received two to three prompts on average over the training period. During the training period, three children dropped out of the study (dropout rate: 4.47%).

All participants revisited the laboratory for a post-intervention assessment, which followed the same procedure as the pre-intervention assessment. After completion of the post-intervention assessment, all participants in the waitlist control group were offered the option of choosing one of the training programs. Participants from all groups were provided with a brief report of pre- and post-intervention assessment results and a summary report of the training *via* mail within 2 months of completing the post-intervention assessment.

### 2.5. Experimental conditions

#### 2.5.1. Intervention group: Yface^4^ (integrated SST program)

Yface is an app-based intervention program designed to enhance the social skills of children with ASD based on research findings on social skills, face perception, and eye gaze. Twelve games, comprising four games per area, were developed by revising and supplementing “Look at Me,” an app-based training developed by Chung et al. (37), and FaceA, a computerized training program developed by Oh and Chung (37). The games are listed in Table 2.

**Table 2:** Description of the 12 games in the Yface training program.

Target area	Name of the game	How to play the game	Difficulty levels
Eye gaze	Gem hunter	Search for a small treasure that appears on the face (predominantly appears near the eyes) as quickly as possible	Size/number/transparency of the gem; duration of stimulus presentation; response time limits
	Stare game	Complete a word by combining the consonants and vowels in the box that the eyes are gazing at	Number of gaze directions; number of syllables in a word; duration of stimulus presentation; response time limits
	Watch my eyes	Among many faces, search for the face with the same eyes as the target eyes presented immediately before	Level of morphing intensity; number of choices; duration of stimulus presentation; response time limits
	Spelling eyes	Guess whether one eye is closed or both eyes are closed	Ratio of presentation for one or both eyes; number of trials; duration of stimulus presentation
Face perception	Find the twins	Memorize multiple faces and find pairs with a different frequency	Number of pairs; level of frequency
	Hi, friend!	Memorize and recognize multiple faces	Number of targets; duration of stimulus presentation; response time limits
	Who was it?	Memorize a front-facing face and search for the same face among many side profiles	Number of choices; duration of stimulus presentation; response time limits
	Catch the thief	After viewing a face stimulus, search for the original face among a selection of photos with altered length of the philtrum or distance between the eyes	Number of choices; duration of stimulus presentation; response time limits
Social skills	Face charades	Find an expression appropriate to the situation among the selection and record yourself reenacting it	Number of choices
	I’m a model	Find a gesture appropriate to the situation among the selection and record yourself reenacting it	Number of choices
	You said, I say	Choose a phrase appropriate for the conversation and record yourself saying it	Level of conversation type
	Storyteller	Rearrange the pictures in the order of various social situations	Number of scenes; response time limits

Six of the 12 games were presented randomly each day to complete them in 2 days. Each game had 15 hierarchical difficulty levels. All participants started at the first level and proceeded to the next one when the correction rate for each level was ≥80%. A multilayered reward system was adopted to stimulate participation and enhance the game performance of the children, such as giving a title—from *Beginner* (lowest) to *Grand Master* (highest)—depending on the performance, receiving points upon daily attendance, and purchasing items in the store to decorate their own space. This training program was based on the Android operating system, and it was assumed that the participants would use their personal smartphones to receive training. Those who did not possess an Android smartphone were provided with one for the study.

#### 2.5.2. Comparison group: Ycog^5^ (a cognitive rehabilitation program)

Ycog is an app-based intervention program designed to enhance executive function in children with neurobehavioral disorders. Ycog comprises 12 games across four areas: inhibition control, working memory, flexibility, and planning. Each game was developed on the basis of a literature review and the previous games used in cognitive rehabilitation programs (Table 3). Other functions, procedures, game stages, and reward systems were identical to those in Yface.

**Table 3:** Description of 12 games in the Ycog training program.

Area of training	Name of the game	How to play the game	Difficulty level
Inhibition control	Let us Fish	Slide the screen in the direction that the fish of a specified color is swimming	Number of fish; number of directions
	Stop or go	Touch the box according to the rules (green background, in the direction of the arrow; yellow background, opposite direction of the arrow; and red background, do not touch)	Duration of the background presentation; valid reaction time; number of directions
	Eat or not	Touch “eat” button for fresh sushi and “trash” button for spoiled sushi	Duration of the sushi presentation; ratio of “eat” and “trash”
	Up and down	Raise, lower, or avoid touching a flag of a certain color according to instructions	Number of flags; number of inhibiting instructions; duration of presentation of flags and instructions
Working memory	Count 123	Memorize numbers in Arabic or Korean and pictures quickly and perform mental calculations	Number of presented numbers; duration of the stimulus presentation
	Find a match	Remember multiple cards and pair the identical pictures	Number of pairs
	Boom clap	Memorize the order of drums being played quickly and play it back	Number of drums played
	Is it there?	Remember the presented words and recognize them	Number of choices; number of targets; duration of word presentation
Flexibility and planning	Tap! Tap! Tap!	Touch the randomly arranged numbers from the smallest to the largest value	Number of presented numbers
	Pair up	Pair items that share a similarity among several items (two pairs are possible)	Number of items presented; number of correct pairs
	Let us go	Choose items people can see in a certain place	Number of choices; number of answers
	Stack up!	Arrange bricks into the presented picture using a minimum number of moves	Number of moves

#### 2.5.3. Control group: waitlist control group

After participants in the waitlist control group completed their pre-intervention assessments, they waited while engaging in their usual activities for the same duration as the two intervention groups (66 days), without further contact with the research team.

## 3. Data analysis

SPSS statistics 21.0 (IBM, Armonk, NY, United States) was used for the data analysis. All dependent variables were normally distributed (Kolmogorov–Smirnov test, >0.05).

The analytical method was as follows. First, as a preliminary analysis, a one-way analysis of variance (ANOVA) was performed as a preliminary analysis to test the differences of the pre-intervention assessment among the three groups. Next, a repeated-measures ANOVA was performed for each dependent variable to evaluate any significant differences across the three groups and over time (pre- and post-intervention). When the group × time interaction effect was found to be significant, the one-way ANOVA of post – pre assessment values was used to examine the difference of each variable among groups. To determine which specific groups showed significant differences, Tukey’s test was conducted as a post-hoc analysis. To determine the effect size, the partial eta squared (*η*^2^) was calculated.

## 4. Results

### 4.1. Testing the group differences in the pre-intervention scores

A one-way ANOVA was performed on the pre-intervention measurements for all dependent variables to test for the differences between the groups. There were no significant differences between the groups in the pre-intervention scores (social responsiveness from SRS, *F*(2, 50) = 0.55, *p* > 0.05; social skills in the interview, *F*(2, 50) = 0.93, *p* > 0.05; face perception in the interview, *F*(2, 50) = 0.00, *p* > 0.05; eye gaze in the interview, *F*(2, 50) = 0.07, *p* > 0.05; dot-probe task*, F*(2, 50) = 0.42, *p* > 0.05).

### 4.2. Effects of the intervention on questionnaire and semi-structured interviews

**Figure 3:**
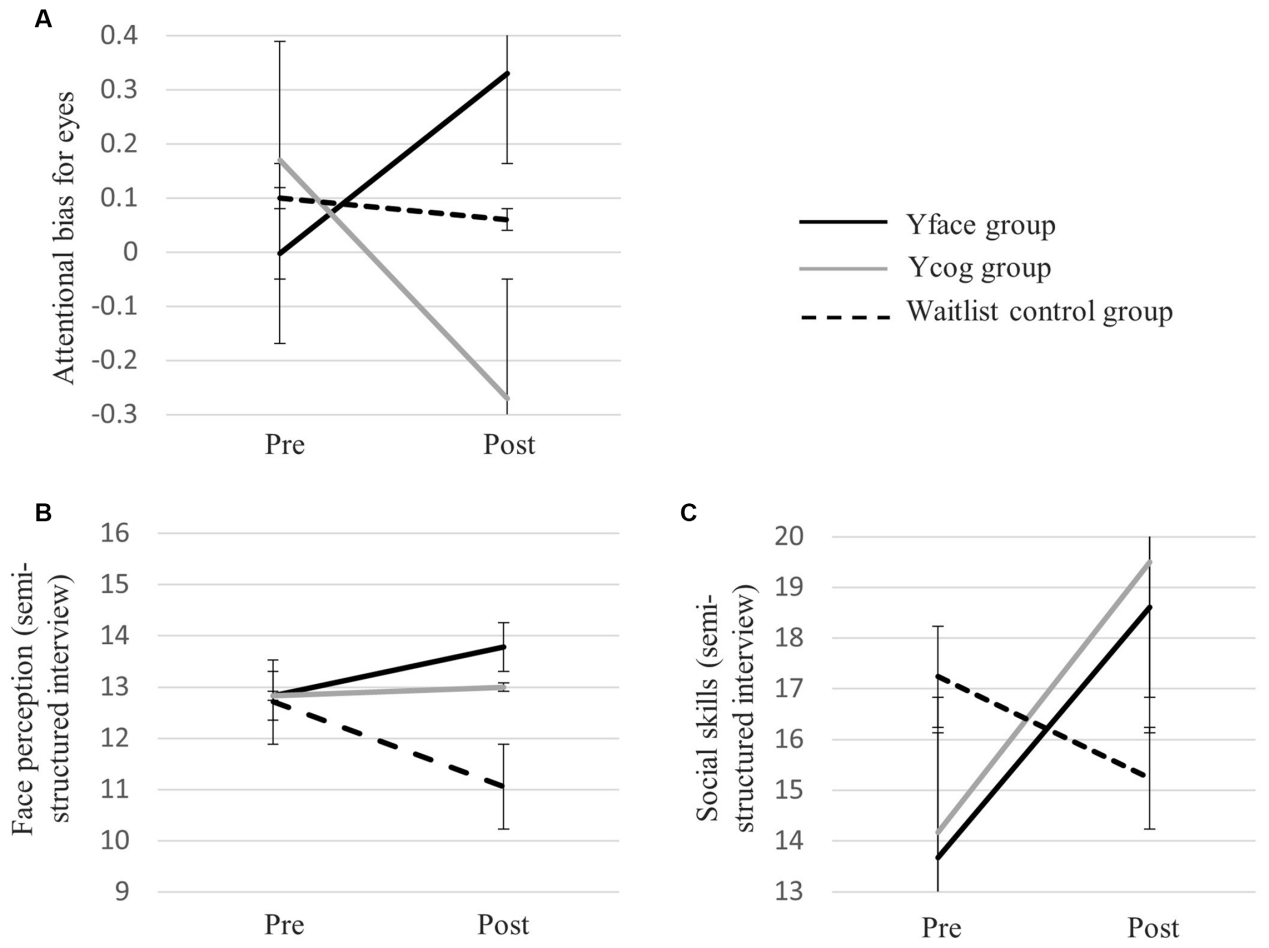
Graphs of the interaction effect of intervention group and time on **(A)** attentional bias for eyes from dot-probe task, **(B)** face perception from semi-structured interview, and **(C)** social skills from semi-structured interview.

A series of repeated-measures ANOVAs were conducted with pre- and post-intervention scores as dependent variables. The mean, standard deviation, and results are presented in Table 4 and Figure 3.

**Table 4:** Repeated-measures ANOVA for pre- and post-intervention scores for each outcome variable by group.

Outcome variable	Time	Yface group (*n* = 18)	Ycog group (*n* = 18)	Waitlist control (*n* = 17)	Source	*F*	*p*-value	Partial *η*^2^
		*M* (SD)	*M* (SD)	*M* (SD)				
Social responsiveness (SRS)	Pre	165.72 (29.09)	158.61 (17.94)	158.24 (23.37)	Group	0.30	0.744	
	Post	152.06 (22.37)	149.11 (19.52)	157.82 (23.96)	Time	8.94**	0.004	0.152
					GxT	2.18	0.123	
Social skills (semi-structured interview)	Pre	13.67 (4.95)	14.17 (7.00)	17.24 (11.84)	Group	0.06	0.943	
	Post	18.61 (6.43)	19.50 (7.29)	15.24 (5.74)	Time	7.65**	0.008	0.133
					GxT	5.59**	0.006	0.183
Face perception (semi-structured interview)	Pre	12.83 (3.93)	12.83 (4.76)	12.71 (3.06)	Group	0.73	0.486	
	Post	13.78 (3.83)	13.00 (4.27)	11.06 (2.88)	Time	0.21	0.652	
					GxT	3.74*	0.031	0.130
Eye gaze (semi-structured interview)	Pre	54.50 (17.01)	55.33 (17.27)	56.53 (15.08)	Group	0.26	0.769	
	Post	61.00 (15.30)	64.17 (13.88)	56.18 (14.33)	Time	6.95*	0.011	0.122
					GxT	2.08	0.135	
Attentional bias for eyes	Pre	0.00 (0.19)	0.17 (0.86)	0.10 (0.32)	Group	0.62	0.543	
	Post	0.33 (0.83)	−0.27 (1.13)	0.06 (0.27)	Time	0.15	0.704	
					GxT	3.57*	0.036	0.134

*p < 0.05. **p < 0.01. *η*^2^ = 0.01 indicates a small effect. *η*^2^ = 0.06 indicates a medium effect. *η*^2^ = 0.14 indicates a large effect.

The results indicate that the interaction between group and time was not significant for social responsiveness (*F*(2, 50) = 2.18, *p* > 0.05). However, the main effect of time was significant (*F*(1, 50) = 8.94, *p* < 0.01, *η*^2^ = 0.152), indicating an improvement in social responsiveness and communication post-intervention for all participants. The main effect of group was not significant (*F*(2, 50) = 0.30, *p* > 0.05).

For social skills, both the interaction effect between group and time (*F*(2, 50) = 5.59, *p* < 0.01, *η*^2^ = 0.183) and the main effect of time (*F*(1, 50) = 7.65, *p* < 0.01, *η*^2^ = 0.133) were significant. The main effect for the group was not significant (*F*(2, 50) = 0.06, *p* > 0.05). The one-way ANOVA and Tukey’s test were performed as a post-hoc analysis to investigate the difference between the post- and pre-intervention scores across the groups. The total score for social skills significantly increased post-intervention in the Yface (*t*(50) = 2.83, *p* < 0.01) and Ycog (*t*(50) = 2.99, *p* < 0.01) groups, compared to the waitlist control group. No significant differences were observed between the two training groups (*t*(50) = −0.16, *p* > 0.05).

A significant interaction between group and time was also observed for face perception (*F*(2, 50) = 3.74, *p* < 0.05, *η*^2^ = 0.130). The post-hoc tests showed that the total score in face perception increased post-intervention in the Yface group compared to the waitlist control group (*t*(50) = 2.67, *p* = 0.01). No significant differences were observed between the Yface and Ycog groups (*t*(50) = 0.81, *p* > 0.05) or between the Ycog and waitlist control groups (*t*(50) = 1.87, *p* > 0.05). The main effects of group (*F*(2, 50) = 0.73, *p* > 0.05) and time (*F*(1, 50) = 0.21, *p* > 0.05) were not significant.

Finally, for eye gaze, both the interaction between group and time (*F*(2, 50) = 2.08*, p* > 0.05) and the main effect of group (*F*(2, 50) = 0.26*, p* > 0.05) were not significant. Only the main effect of time was significant (*F*(2, 50) = 6.95, *p* < 0.05, *η*^2^ = 0.122), indicating an improvement in the parent-reported eye gaze post-intervention for all participants.

### 4.3. Effects of the intervention on the dot-probe task

To determine a significant difference in attentional bias for eyes pre- and post-intervention across the groups, a repeated-measures ANOVA was performed with the attentional bias score as the dependent variable. The pre-and post-intervention measurements for each task by the group and the results are presented in Table 4.

A significant interaction between group and time was observed (*F*(2, 46) = 1.22, *p* < 0.05, *η*^2^ = 0.134). A one-way ANOVA and Tukey’s test as post-hoc testing showed that attentional bias for eyes increased significantly in the Yface group compared to the Ycog group (*t*(46) = 2.67, *p* = 0.01). No significant difference was found between the Yface and waitlist control groups (*t*(46) = 1.30, *p* > 0.05), or between the Ycog and waitlist control groups (*t*(46) = −1.35, *p* > 0.05). The main effects of group (*F*(2, 46) = 0.62, *p* > 0.05) and time (*F*(1, 46) = 0.15, *p* > 0.05) were not significant.

## 5. Discussion

An app-based integrated program, Yface, which combines social skills, face perception, and eye gaze training, was developed, and its effectiveness was pilot tested on a sample of 53 children with ASD (aged 7–15 years) for 66 days, as compared to those of a waitlist control group and a group that completed cognitive rehabilitation training using another app, Ycog. The results indicated significant improvements in social skills and face perception in the Yface group compared to the waitlist control group. In addition, the Yface group showed significant improvements in attentional bias for eyes compared to the Ycog group. There were no significant differences between the groups in terms of social responsiveness or eye gaze. The research and clinical implications of this study are as follows.

First, the Yface program was found to be effective in improving eye gaze, face perception, and social skills, which is consistent with the practice-makes-improvement learning theory. Other social skills training programs have reported similar results in individuals with ASD (33, 34). Interestingly, significant improvement was observed in all areas regardless of the training type or outcome measures. The training time for each area was similar as an equal number of games were presented across all three areas at a comparable rate. This suggests that a similar amount of training time or practice is needed to make progress in all three areas, but further investigation is needed to compare the relative difficulties of acquiring skills in these areas. Moreover, positive effects were found in both subjective and objective outcome measures, which provides stronger evidence of the effectiveness of the Yface program. Overall, these findings are useful for designing effective training programs for individuals with ASD, as they suggest that a similar number of tasks are needed regardless of the training area, and that a combination of subjective and objective outcome measures can provide stronger evidence of training effectiveness.

However, the study did not support the hypothesis that the training effect would generalize to overall social ability. This is rather an unexpected results, since previous studies (36, 37) have reported that a combined training program for eye gaze and face perception resulted in improvements in targeted areas and overall social skills. One explanation could be the brief training period of 66 days, highlighting the need for further investigations on the optimal duration and intensity of training. Additionally, the study only assessed social responsiveness as a measure for social ability, so future studies should include measures investigating other areas of social ability.

Despite its significance, caution must be exercised when interpreting the current results. First, although this training program showed overall improvements in social interaction, it was limited in some respects. For example, the Yface group showed improved social skills and face perception relative to the waitlist control group, but not compared to the Ycog group. This may result from the improvement found in the cognitive rehabilitation training group, which implies that an overall enhancement in social skills may be associated with improved executive function. For example, cognitive flexibility and behavioral control are key predictors of social ability development (50), and play a critical role in the effective use of social skills in daily life (51). Furthermore, working memory capacity may be a mechanism underlying social skills, and is an essential factor in using social norms and controlling behaviors in complex social situations (52). These results suggest that the enhancement of cognitive function due to cognitive rehabilitation led to improved social skills. The positive effects of cognitive rehabilitation training clearly show the need for a follow-up study on the role and mechanism of executive functioning in improving social skills in children with ASD, and for considering the inclusion of cognitive function training as an alternative to (or a part of) SST. Second, no interaction between group and time was found for social responsiveness or eye contact. In addition, attentional bias for eyes increased significantly in the Yface group compared to the Ycog group, but not compared to the waitlist control group, which may indicate no improvement in eye gaze using the Yface app compared to no treatment. One reason may be the wide age range of the participants (7–15), from school-age children to adolescents, included in this pilot study. They often have different needs and developmental tasks, in terms of social skills and demands in the real world (53). Further studies need to separate adolescents (12 years or above) and pre-adolescent school-aged children (6–11) to examine the effectiveness of Yface in each target age.

As a new attempt to combine diverse training approaches, this study reaches beyond the limitations of existing studies, in terms of training areas and social validity. While the generalization of treatment effects is a major concern in treatment outcome research, the social validity of technology-based training has been taxed by the discrepancy between the training medium and its application in real-life settings (54). Our positive results will inform the development of an effective, efficient, and socially acceptable treatment modality.

Second, this study demonstrated the utility of technology-based interventions as an alternative to traditional psychotherapy in children with ASD. The most effective treatment for ASD is applied behavior analysis (55), which involves the acquisition of necessary skills through repetition with the help of a therapist. Our results show that some ASD symptoms may be partially improved through repetitive training using devices without the help of professionals. Although various forms of app- or web-based cognitive-behavioral therapy are effective for many clinical groups (56–58), technology-based interventions have not been thoroughly tested in children from diverse clinical populations, to the best of our knowledge. In particular, children do not have much interest in or motivation to improve symptoms, compared to adults (59). Thus, strategies that enable children to independently and voluntarily engage in training without help or supervision are essential for technology-based interventions to be effective.

Gamification (60), which is the concept of applying successful game elements to non-game areas, has been actively integrated into this training program from the design stage of development. Examples include the diversification of themed rewards, adoption of items favored by children with ASD, implementation of a level system based on performance, and visual and auditory reinforcements based on performance. This strategy was deemed to be successful: not only was the dropout rate very low during the ~66 days of training (4.47%), but several participating children expressed their continual interest in specific games or program components to research team members throughout the training. This was encouraging not only for facilitating children’s engagement in the treatment program, but also for lessening the parental burden related to their children’s non-compliance with treatment (61). In addition, training using smartphone apps has a major advantage as a long-term training device that taps into children’s interests, reduces fatigue and boredom, and increases self-motivation (62, 63). Furthermore, an app-based intervention program has high accessibility and low cost, highlighting its utility among populations with ASD.

Third, this study is significant in testing the effect of an intervention on the social skills of children with ASD using a rigorous scientific method, namely, randomized controlled trials (RCTs). Among the existing studies on technology-based interventions to improve social skills in children with ASD (64, 65), studies that use RCTs are very rare. Most studies in the field utilize quasi-experimental designs, such as one-group pre/post-test and group designs without random assignment, or single case designs, rather than RCTs (66–68). The lack of RCT studies was noted as a significant limitation by Wong et al. (69), who thoroughly reviewed evidence-based ASD treatments. Technology-based intervention studies based on RCTs similar to the present study should be conducted in the future.

There are several limitations to this study, and follow-up studies would be advisable. First, it should be noted that most of our participants were recruited from the Seoul Metropolitan area, of which, 96% were boys. This may restrict the generalizability of our findings to a more diverse range of children with ASD. Therefore, a replication study with a more gender-balanced sample and diverse demographic characteristics is a critical step toward developing an evidence-based intervention model. Second, only children with high-functioning ASD participated in this study because they were required to independently operate the smartphone app and play the games. Several participants were excluded from the screening process because they did not meet the IQ criteria. For technology-based interventions to be applied to the ASD population more comprehensively, a program with several difficulty levels is needed. Third, generalization of training was not studied, mainly owing to the lack of appropriate measures to test the generalization of our findings to daily life, which limits the interpretability of our findings. In addition, no long-term follow-up was performed in this study, thus limiting the generalizability of the results. Investigations of the long-term effects of training in various social situations are needed. Fourth, a semi-structured interview was developed and administered in this study because of the absence of an assessment instrument that measures eye gaze, face perception, and social skills. However, validity was not calculated in the study by comparison with a group of typically developing children. Thus, it will be necessary to evaluate the validity and reliability of the developed instrument in future studies. Finally, as this study is a pilot study, a rigorous replication is needed. This study is the first to test combined training in three specific skills (eye gaze, face perception, and social skills) to improve the social skills of children with ASD. Therefore, replication studies are required to retest and consolidate the effectiveness of integrated training and complement the Yface program using their results. Despite that sample size of this study was larger than the recommended number from the post-hoc power analysis (53 versus 42, respectively), a larger sample size would be needed for a more rigorous replication.

## 6. Conclusion

Our results demonstrate the possibility of greater benefits to providing integrated training that covers multiple social domains, rather than focusing solely on a single domain like the traditional SST that is often used for children with ASD. Furthermore, our study demonstrates the potential use of technology-based training as an alternative or assisted intervention for ASD. Specifically, our findings suggest that perceptual training for eye gaze and face perception, which has been challenging to implement in traditional SST, can be easily accomplished through the use of technology. Especially since the COVID-19 pandemic, technology-based health interventions like the one used in our study have become increasingly important in the provision of ASD treatment services. Therefore, it is crucial that efforts are made to actively promote the development and dissemination of more accessible and effective programs in the future. On the basis of this pilot study, technology-based interventions for improving integrated social abilities in children with ASD should be developed. Furthermore, there is a need for more systematic research, including RCTs, to verify the effectiveness of such interventions.

## Data availability statement

The raw data supporting the conclusions of this article will be made available by the authors, without undue reservation.

## Ethics statement

All procedures were performed in accordance with the ethical standards of the Yonsei University Institutional Review Board (no. 7001988-201712-SB-253-10) and with the 1964 Helsinki Declaration and its later amendments or comparable ethical standards. Written informed consent to participate in this study was provided by the participants’ legal guardian/next of kin. Written informed consent was obtained from the individual(s) for the publication of any identifiable images or data included in this article.

## Author contributions

KC developed the theoretical framework and designed the experiments. EC performed the experiments, analyzed the data, and interpreted the results. KC and EC wrote the article. All authors contributed to the article and approved the submitted version.

## Funding

This research was supported by a grant from the Korea Health Technology R&D Project through the Korea Health Industry Development Institute, funded by the Ministry of Health & Welfare, Republic of Korea (no. HI15C0817).

## Conflict of interest

The authors declare that the research was conducted in the absence of any commercial or financial relationships that could be construed as a potential conflict of interest.

## Publisher’s note

All claims expressed in this article are solely those of the authors and do not necessarily represent those of their affiliated organizations, or those of the publisher, the editors and the reviewers. Any product that may be evaluated in this article, or claim that may be made by its manufacturer, is not guaranteed or endorsed by the publisher.

## Footnotes

^1^ABA is a scientific approach to understanding behavior and how it is affected by the environment based on the principles of behaviorism. ABA uses a systematic approach to identifying and analyzing behavior, determining the factors that influence it, and developing interventions to modify it (6, 7).

^2^This study was supported by Samsung Electronics. The program is no longer available; however, an article and video clip introducing the project can be found at https://design.samsung.com/kr/contents/lookatme/..

^3^A licensed clinical psychologist (Board Certified Behavior Analyst–Doctoral).

^4^The Korean and English demo and main versions of Yface can be downloaded from the Google Play store. To download the application, search "Yface" at the Google Play store or use the following link: https://play.google.com/store/apps/details?id=yonseipsychology.yface. This game may be used after consenting to participate in the study and completing a questionnaire.

^5^The Korean and English demo and main versions of Ycog can be downloaded from the Google Play store. To download the application, search "Ycog" at the Google Play store or use the following link: https://play.google.com/store/apps/details?id=yonseipsychology.ycog. This game may be used after consenting to participate in the study and completing a questionnaire.
